# Survival for endometrial cancer as a second primary malignancy

**DOI:** 10.1002/cam4.4554

**Published:** 2022-01-30

**Authors:** Heidy N. Medina, Matthew P. Schlumbrecht, Frank J. Penedo, Paulo S. Pinheiro

**Affiliations:** ^1^ Department of Public Health Sciences University of Miami School of Medicine Miami Florida USA; ^2^ Sylvester Comprehensive Cancer Center University of Miami School of Medicine Miami Florida USA; ^3^ Department of Obstetrics & Gynecology University of Miami School of Medicine Miami Florida USA; ^4^ Department of Psychology University of Miami Miami Florida USA

**Keywords:** cancer survivors, cause‐specific, endometrial cancer, second primary, survival

## Abstract

**Background:**

Endometrial cancer (EC) often occurs subsequently to a primary cancer arising from a different site. However, little is known regarding the survival experience of EC as a second primary (ECSP) malignancy, specifically in relation to the original primary site and prior treatment.

**Methods:**

Using Florida's cancer registry, all EC cases (first, second, or higher‐order) diagnosed from 2005–2016 were analyzed. Kaplan–Meier methods and Cox Regression were used in a cause‐specific survival analysis.

**Results:**

A total of 2879 clinically independent ECSPs and 42,714 first primary ECs were analyzed. The most common first primary sites for ECSPs were breast cancer (BC) (*n* = 1422) and colorectal cancer (CRC) (*n* = 359). Five‐year cause‐specific survival was 84.0% (95% CI: 83.6–84.3) for first primary ECs and 81.8% (95% CI: 80.0–83.4) for ECSPs. After adjusting for age, race/ethnicity, histology, and stage at diagnosis, ECSPs had a lower risk of EC mortality than first primary ECs (hazard ratios [HR] 0.88, 95% CI: 0.79–0.97). ECSPs with a first primary CRC had a higher risk of EC‐specific death (HR 1.47, 95% CI: 1.04–2.06) compared to ECSPs that followed BC in multivariable analysis. Finally, women who had chemotherapy for ECSP and preceding BC did not have a higher risk of death (HR 0.80, 95% CI: 0.49–1.31) compared to those who only received chemotherapy for first primary EC.

**Conclusions:**

ECSPs present a complex clinical profile. ECSP survival is superior to that of first primary EC. However, ECSPs following CRC may constitute a population of interest for their worse prognosis. Chemotherapy for a previous BC does not seem to impact the effectiveness of chemotherapy for ECs.

## Graphical abstract

Second primary endometrial cancers are of worse histological type and present at a more advanced stage at diagnosis. Despite this, endometrial cancer as a second primary has improved survival in relation to first primary endometrial cancers after taking into account these tumor‐related characteristics. Endometrial cancer after a primary colorectal cancer has worse prognosis than following a primary breast cancer.

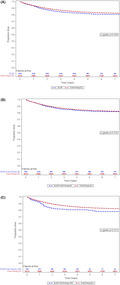

## 1 INTRODUCTION

Second primary malignancies account for nearly 20% of all new cancer cases^1^ and are a main cause of morbidity and mortality among cancer survivors.^2^ In comparison to the general population, cancer survivors have a higher risk of developing and dying from a new cancer.^3^ Moreover, advances in screening and surveillance of cancer survivors have also contributed to improvements in the identification of cases of second primary tumors.^4^, ^5^ In addition to the common risk factors involved in the development of first primary tumors such as lifestyle factors (e.g., tobacco, alcohol, diet) and environmental exposures (e.g., contaminants, occupation), important considerations for the development of second primary tumors include a younger age at diagnosis, cancer site, and prior treatment modality for the first cancer.^4^, ^5^

Currently, obesity‐related cancers such as endometrial cancer (EC) account for a considerable proportion of second primary tumors.^3^ EC is a common second primary malignancy after breast (BC), ovarian, cervical, and colorectal (CRC) cancer.^4^, ^5^, ^6^, ^7^, ^8^, ^9^ Prior chemotherapy and hormonal therapy for BC is associated with an increased risk of subsequent EC^6^, ^10^, ^11^; for postmenopausal women who use tamoxifen, specifically, there is a two to four‐fold elevated risk of EC as a second primary (ECSP).^12^ Women who receive pelvic radiotherapy for a first primary rectal or cervical cancer are also more likely to develop EC,^13^, ^14^ on average 14 years later in the case of cervical cancer.^14^ Additionally, early‐onset second primary cancers such as EC may be indicative of hereditary cancer syndromes such as Lynch Syndrome, with women having a cumulative lifetime risk of 40%–60% of developing EC.^15^ Past research has also demonstrated an increased risk of EC among *BRCA* carriers, but this has been partly attributed to prior tamoxifen treatment for BC.^16^

Second primaries are an unavoidable problem as the number of cancer survivors and size of the aging population inevitably increases. The theme of EC as a second primary has hardly been explored. Previous studies have focused on examining second primary malignancies after a first primary of EC^7^, ^17^, ^18^, ^19^, ^20^, ^21^ and the clinicopathological features of ECSP.^22^, ^23^ Little is known regarding ECSP survival on a population basis, in general, and in regard to the specific aspects of the primary cancer site and prior treatment. Therefore, the objectives of this study are to examine the clinical/demographic distribution of ECSPs, the survival differences between ECSPs and ECs as a first primary, and to analyze the effect of prior chemotherapy for BC on survival for chemotherapy‐treated ECSPs.

## 2 METHODS

### 2.1 Data source and population

All ECs diagnosed between 2005 and 2016 in Florida were identified from the Florida Cancer Data System (FCDS) according to the *International Classification of Disease, for Oncology, 3rd edition (ICD‐O‐3)* topography site codes C54.X and C55.9 and morphology codes 8000–8951.^24^ FCDS is the statewide cancer registry for Florida and has been nationally certified by the North American Association of Central Cancer Registries (NAACCR) at its highest level for meeting standards for completeness, timeliness, and quality with overall completeness >95%.^25^ For attribution of second or higher‐order ECs, as opposed to EC as a first primary, data for first primary cancers of all sites diagnosed between January 2000 and December 2016 were abstracted. A first primary EC only was defined as those with a sequence number of 0 (one primary only) and 1 (first primary of two or more primaries) while those with a sequence number of 2 or above (second or higher‐order primary of two or more primaries) were defined as an ECSP. For ECSPs, the corresponding first primary cancer site was considered to be the one identified as a sequence number of 1 only.

Histologic subtypes of EC were categorized according to Cote et al.^26^ into clear cell (8310), endometrioid (8050, 8140, 8143, 8210–8211, 8260–8263, 8340, 8380–8384, 8560, 8570), mixed cell (8255, 8323), malignant Mullerian mixed tumors (MMMT) and carcinosarcomas (8950–8951, 8980–8981), serous (8441, 8460–8461), and other (neuroendocrine [8013, 8041, 8045–8046, 8574], undifferentiated [8020], endometrioid with unknown grade, and general histologic descriptions). As in previous research, endometrioid low‐grade carcinomas were considered EC Type I, while the remaining were categorized into EC Type II.^27^, ^28^, ^29^ The worse prognosis for EC Type II in relation to EC Type I has been extensively documented.^26^, ^27^, ^28^, ^29^, ^30^, ^31^ Surveillance, Epidemiology and End Results (SEER) stage were considered as established in registry‐based analyses; they mirror FIGO staging as follows: localized (FIGO IA, IB, IC, and FIGO stage I not further specified), regional (FIGO stage IIA, IIB, or FIGO stage II, not otherwise specified, FIGO stage IIIA, IIIB, and IIIC), distant (FIGO stage IVA, IVB), and unknown.^26^, ^32^

Sociodemographic and tumor‐related variables including age, race/ethnicity, receipt of chemotherapy, and essential follow‐up data (date and cause of death) were abstracted from FCDS. Race/ethnicity was classified as non‐Hispanic White, non‐Hispanic Black, non‐Hispanic Asian and Pacific Islander, and Hispanics of any race, hereby referred to as White, Black, API, and Hispanic, respectively, for simplicity. For ECSPs, all relevant sociodemographic and clinical characteristics for the corresponding first primary cancer site were also compiled.

Registries follow standard NAACCR coding rules in handling multiple tumors.^33^ These rules include the following: a cancer of a different site and histologic type than the original cancer is a separate primary; cancers of a different family of histologic types in the same site are separate primaries regardless of when they are diagnosed; a new diagnosis of a malignancy in the same site and histology as a previous diagnosis is considered the same primary cancer if diagnosed within 2 months or a separate primary cancer if diagnosed after 2 months. For the purpose of our study, we considered clinically independent ECSPs only. These were defined as cases that would require different gynecological treatments of curative intent for each of the first and second primaries. In accordance with this, those ECSPs with first primary ovarian or cervical cancer diagnosed within 1 year were excluded, and those ECSPs with a first primary of EC with the same or similar histology were considered to be a first primary EC instead.

### 2.2 Statistical analyses

We examined frequency distributions for all clinical and sociodemographic characteristics for first primary ECs and ECSPs and used chi‐square tests to determine differences between the two. Patients diagnosed with sarcomas of the corpus uterus and gestational trophoblastic tumors (*n* = 2253), those who had negative survival time (*n* = 48), and those diagnosed at autopsy or by death certificate only (*n* = 327) were excluded from the analyses. Cause‐specific survival was defined as the elapsed time in days from the date of disease diagnosis to the date of death, or the date of last mortality linkage, December 31, 2016, whichever occurred first. The outcome was based on the specific cause of death, EC; therefore, survival calculations for deaths from other causes were censored at the time of death. Cause‐specific, 5‐year survival for first primary EC and ECSP was estimated using the Kaplan–Meier method with corresponding 95% confidence intervals. We used the log‐rank test to test univariable differences. Hazard ratios (HR) were calculated using the Cox‐proportional regression analysis in order to assess multivariable differences in overall EC‐specific survival, by ECSP and EC as a first primary, and corresponding primary cancer site (for ECSPs). Within those with ECSP, models examined the main effect of the first primary cancer site as well as chemotherapy treatment (for those with first primary BC only). Multivariable models were adjusted for age, race/ethnicity, histology subtype, and stage at diagnosis (of EC and first primary cancer site for ECSPs). Proportional hazards assumptions were assessed by inspecting the correlation between scaled Schoenfeld residuals and survival time and testing the time‐dependent covariates for each model.

A cause‐specific Cox‐proportional hazards survival approach was chosen as it allowed us to examine the survival of patients with first primary EC and ECSPs, in the theoretical scenario in which EC would be the only cause of death. In previous literature, Howlader et al. and Mariotto et al. explain that cause‐specific survival is the best measure to compare groups of cancer patients (such as patients with first primary EC vs. ECSPs) and evaluate the impact of clinical determinants and treatment on cancer survival.^34^, ^35^ Cause‐specific survival approaches are ideal to answer questions related to health policy, research, and biology, while competing risk analyses are preferred in the context of real‐life survival probabilities, prediction models, and clinical decision making.^34^, ^35^

Analyses were performed in SAS University Edition. All *p*‐values were reported as 2‐sided, with statistical significance defined as *p* < 0.05. The study was approved by the Florida Department of Health Institutional Review Board.

## 3 RESULTS

**FIGURE 1:**
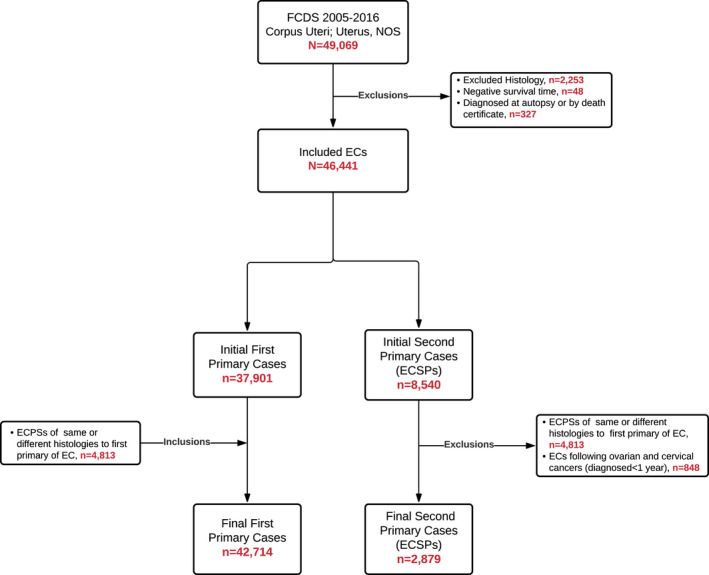
Identification of EC cases according to first primary EC and ECSP using FCDS. EC, endometrial cancer; ECSP, endometrial cancer as a second primary

There were 46,441 diagnosed cases of EC initially considered for analysis during 2005–2016. Of these, 37,901 were first primary cases and 8540 were second or higher‐order primary cases. Among those identified as a second or higher‐order primary, 9.9% (*n* = 848) were ECSPs with first primary ovarian or cervical cancer diagnosed within 1 year (representing 93.5% of all ECSPs with first primary ovarian or cervical cancer in general) and 56.4% (*n* = 4813) were ECSPs with a first primary of EC with same or similar histology. After both of these exclusions, a total of 45,593 cases were included for analysis, of which 42,714 (93.7%) were first primary ECs and 2879 (6.3%) were ECSPs (Figure 1). The median age at diagnosis for first primary ECs and ECSPs were 65 and 69 years old, respectively. Those with first primary ECs had a higher proportion of Type I, low‐grade endometrioid (57.8% vs. 46.3% for ECSPs) while ECSPs had a higher proportion of Type II ECs (38.2% vs. 29.2% for first primary ECs) (both*, p* < 0.0001). ECSPs had a higher proportion for carcinosarcoma (8.8%), clear cell (2.3%), mixed cell (4.7%), and serous EC (10.6%) in comparison to EC as a first primary (5.6%, 1.6%, 3.4%, and 5.9%, respectively) (all, *p <* 0.01). ECSPs were more likely to be diagnosed at a distant stage, 9.0% vs. 7.4% for first primary ECs (*p <* 0.01). The most commonly diagnosed first primary sites among ECSPs were BC and CRC (Table 1), with a median age of ECSP diagnosis at 69 and 72 years old, respectively (Table S1). There was a median time of 49 and 44 months between the diagnosis of the first primary BC and CRC, respectively, and subsequent ECSP. Other most common primary sites in descending order included: skin melanoma, lung, bladder, thyroid, kidney, oral cavity and pharynx, soft tissue sarcoma, and ovary.

**TABLE 1:** Clinical and demographic characteristics of first primary ECs and ECSPs, Florida 2005–2016

	Total *n* (%)	First primary EC n (%)	ECSP *n* (%)	*p‐*value^a^
Total	45,593 (100.0%)	42,714 (93.7%)	2879 (6.3%)	
Age category				<0.0001
15–44	2506 (5.5%)	2449 (5.7%)	57 (2.0%)	
45–54	6190 (13.6%)	5910 (13.8%)	280 (9.7%)	
55–64	13,628 (29.9%)	12,970 (30.4%)	658 (22.9%)	
65–74	13,460 (29.5%)	12,557 (29.4%)	903 (31.4%)	
75+	9807 (21.5%)	8827 (20.7%)	980 (34.1%)	
Race/ethnicity				<0.0001
White	33,129 (72.7%)	30,900 (72.3%)	2229 (77.4%)	
Black	5173 (11.4%)	4896 (11.5%)	277 (9.6%)	
Hispanic	6256 (13.7%)	5925 (13.9%)	331 (11.5%)	
API	533 (1.2%)	505 (1.2%)	28 (1.0%)	
Other	502 (1.1%)	488 (1.1%)	14 (0.5%)	
Histology/type				<0.0001
Type I				
Low‐grade endometrioid	26,010 (57.1%)	24,676 (57.8%)	1334 (46.3%)	
Type II				
All histologies combined	13,553 (29.7%)	12,452 (29.2%)	1101 (38.2%)	
High‐grade endometrioid	5724 (12.6%)	5382 (12.6%)	342 (11.9%)	
Carcinosarcoma	2662 (5.8%)	2409 (5.6%)	253 (8.8%)	
Clear cell	735 (1.6%)	669 (1.6%)	66 (2.3%)	
Mixed cell	1594 (3.5%)	1460 (3.4%)	134 (4.7%)	
Serous	2838 (6.2%)	2532 (5.9%)	306 (10.6%)	
Other	6030 (13.2%)	5586 (13.1%)	444 (15.4%)	
Stage				0.001
Localized	27,146 (59.5%)	25,479 (59.7%)	1667 (57.9%)	
Regional	9685 (21.2%)	9041 (21.2%)	644 (22.4%)	
Distant	3403 (7.5%)	3144 (7.4%)	259 (9.0%)	
Unknown	5359 (11.8%)	5050 (11.8%)	309 (10.7%)	
Primary site				–
Breast	–	–	1422 (49.4%)	
Colorectal	–	–	359 (12.5%)	
Skin melanoma			190 (6.6%)	
Lung	–	–	106 (3.7%)	
Bladder	–	–	81 (2.8%)	
Thyroid			77 (2.7%)	
Kidney	–	–	67 (2.3%)	
Oral cavity and pharynx			42 (1.5%)	
Soft tissue sarcoma			41 (1.4%)	
Ovary^b^			30 (1.0%)	
Other	–	–	464 (16.1%)	
Sequence number				–
2	–	–	2054 (71.3%)	
3	–	–	653 (22.7%)	
4	–	–	130 (4.5%)	
5	–	–	37 (1.3%)	
6	–	–	5 (0.2%)	

Abbreviations: EC, endometrial cancer; ECSP, endometrial cancer as a second primary.

^a^ *p‐value* from chi‐square test.

^b^ ECSPs with first primary ovarian cancer diagnosed within 1 year were excluded.

**FIGURE 2:**
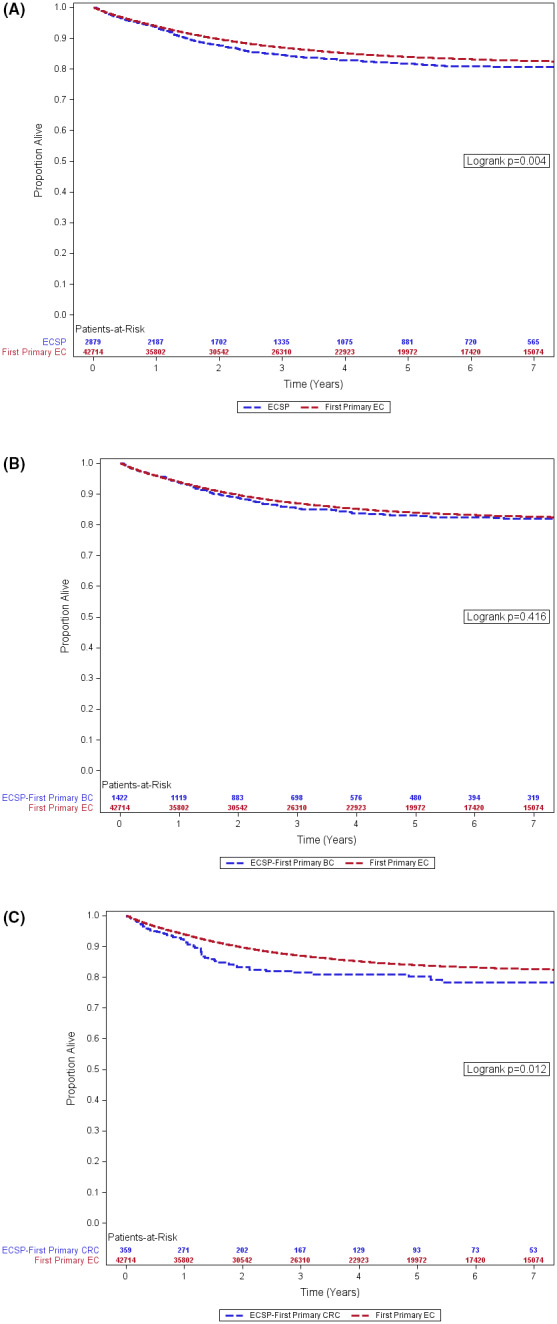
(A) Kaplan‐Meier survival, first primary EC vs. ECSP. (B) Kaplan‐Meier survival, first primary EC vs. ECSP with first primary BC. (C) Kaplan‐Meier survival, first primary EC vs. ECSP with first primary CRC. Florida 2005–2016. BC, breast cancer; CRC, colorectal cancer; EC, endometrial cancer; ECSP, endometrial cancer as a second primary

Overall, Kaplan–Meier EC‐specific, 5‐year survival was 84.0% (95% CI:83.6–84.3) for first primary ECs and 81.8% (95% CI: 80.0–83.4) for ECSPs (Figure 2A). There was an observed difference in EC‐specific survival between first primary ECs and ECSPs (log‐rank test *p* = 0.004), with ECSPs showing a lower survival in comparison to first primary ECs (Figure 2A). Figure 2B shows no observed difference in cause‐specific survival between ECSPs following a first primary BC and those with EC as a first primary (log‐rank test *p* = 0.416). In Figure 2C, there was a significant difference in EC‐specific survival comparing ECSPs with a first primary CRC to first primary ECs (log‐rank test *p* = 0.012), with a lower survival for ECSPs with antecedent CRC.

In multivariable Cox‐proportional hazard analyses, after adjusting for age, race/ethnicity, histology, and stage at diagnosis, ECSPs had a 12% lower risk (HR 0.88; 95% CI: 0.79–0.97; *p* = 0.012) of EC‐specific death compared to first primary ECs (Table 2). ECSPs following a first primary BC had a lower risk of death (HR 0.84; 95% CI 0.73–0.97; *p* = 0.018) than first primary ECs. There was no significant difference in risk of EC‐specific death between ECSPs with a first primary CRC and those with EC as a first primary.

**TABLE 2:** EC‐specific survival^a^ for first primary ECs and ECSPs, overall and by most common primary cancer sites, Florida 2005–2016

	Multivariable^b^	
	HR (95% CI)	*p‐value*
First primary EC (*n* = 42,714)	REF	
ECSP (*n* = 2879)	0.88 (0.79–0.97)	0.012
		
First primary EC (*n* = 42,714)	REF	
ECSP (first primary BC) (*n* = 1422)	0.84 (0.73–0.97)	0.018
		
First primary EC (*n* = 42,714)	REF	
ECSP (first primary CRC) (*n* = 359)	1.08 (0.83–1.39)	0.574

Abbreviations: BC, breast cancer; CRC, colorectal cancer; EC, endometrial cancer; ECSP, endometrial cancer as a second primary; HR, hazard ratio.

^a^ Hazard ratios obtained from Cox‐proportional hazards regression.

^b^ Model adjusted for age category, race/ethnicity, histology, and stage at diagnosis.

Table 3 shows the differences in EC‐specific survival when stratified by first primary ECs and ECSPs. First primary EC older age‐age groups (i.e., 55–64, 65–74, 75+) had a higher risk of cause‐specific death in comparison to those 15–44 years old. Older women with ECSPs had a similar risk of EC‐specific death in relation to those that were younger. For first primary EC, Black women had a 34% higher risk of EC‐specific death (HR 1.34; 95% CI: 1.25–1.44; *p* < 0.0001), and Hispanic women had a 14% lower risk (HR 0.86; 95% CI 0.79–0.93; *p* = 0.0002) than White women. For ECSPs, there were no significant differences in survival by race/ethnicity. First primary Type II ECs had about 3.4 times greater risk of EC‐specific death compared to those with Type I (HR 3.39; 95% CI: 3.16–3.63; *p* < 0.0001) while ECSPs of Type II had a 4.6 times higher risk of mortality than Type I ECSPs (HR 4.55; 95% CI: 3.38–6.13; *p* < 0.0001). ECSPs and first primary ECs diagnosed at regional stage, alike (first primary EC: HR 3.58, 95% CI 3.33–3.83; ECSP: HR 3.12, 95% CI 2.40–4.07), had a higher risk of cause‐specific death than those diagnosed at a localized stage. ECs as a first primary diagnosed at distant stage in relation to localized had a more exacerbated risk of death (HR 12.14; 95% CI 11.24–13.11) than observed for ECSPs (HR 7.38; 95% CI 5.47–9.96).

**TABLE 3:** Demographic and clinical prognostic factors of EC‐specific survival by first primary EC and ECSPs, Florida 2005–2016

Prognostic factors	First primary EC		ECSP	
	HR (95% CI)	*p‐value*	HR (95% CI)	*p‐value*
Age category				
15–44 (*n* = 2506)	REF		REF	
45–54 (*n* = 6190)	1.20 (0.99–1.45)	0.058	0.64 (0.25–1.62)	0.348
55–64 (*n* = 13,628)	1.79 (1.51–2.13)	<0.0001	1.02 (0.45–2.35)	0.959
65–74 (*n* = 13,460)	2.29 (1.93–2.72)	<0.0001	1.16 (0.51–2.65)	0.730
75+ (*n* = 9807)	3.17 (2.67–3.77)	<0.0001	1.80 (0.80–4.09)	0.158
Race/ethnicity				
White (*n* = 33,129)	REF		REF	
Black (*n* = 5173)	1.34 (1.25–1.44)	<0.0001	1.26 (0.95–1.68)	0.105
Hispanic (*n* = 6256)	0.86 (0.79–0.93)	0.0002	1.13 (0.84–1.51)	0.428
API (*n* = 533)	0.84 (0.65–1.09)	0.180	0.66 (0.09–4.76)	0.683
Other (*n* = 502)	0.89 (0.66–1.19)	0.419	1.93 (0.72–5.20)	0.192
Histology				
Type I				
Low‐grade endometrioid (*n* = 26,010)	REF		REF	
Type II				
All histologies combined (*n* = 13,553)	3.39 (3.16–3.63)	<0.0001	4.55 (3.38–6.13)	<0.0001
High‐grade endometrioid (*n* = 5724)	3.22 (2.98–3.49)	<0.0001	3.87 (2.70–5.54)	<0.0001
Carcinosarcoma (*n* = 2662)	5.21 (4.77–5.69)	<0.0001	7.56 (5.37–10.65)	<0.0001
Clear cell (*n* = 735)	2.84 (2.40–3.36)	<0.0001	2.90 (1.60–5.27)	0.0005
Mixed (*n* = 1594)	2.40 (2.10–2.74)	<0.0001	4.19 (2.66–6.60)	<0.0001
Serous (*n* = 2838)	3.04 (2.77–3.35)	<0.0001	3.80 (2.65–5.45)	<0.0001
Other (*n* = 6030)	2.73 (2.49–2.98)	<0.0001	2.62 (1.79–3.85)	<0.0001
Stage				
Localized (*n* = 27,146)	REF		REF	
Regional (*n* = 9685)	3.58 (3.33–3.83)	<0.0001	3.12 (2.40–4.07)	<0.0001
Distant (*n* = 3403)	12.14 (11.24–13.11)	<0.0001	7.38 (5.47–9.96)	<0.0001
Unknown (*n* = 5359)	3.88 (3.51–4.28)	<0.0001	4.48 (3.09–6.48)	<0.0001

Abbreviations: EC, endometrial cancer; ECSP, endometrial cancer as a second primary; HR, hazard ratio.

Lastly, among ECSPs exclusively, those following CRC had a significantly higher risk of EC‐specific death (HR 1.47; 95% CI: 1.04–2.06; *p* = 0.028) compared to those with first primary BC (Table 4). Women who had chemotherapy for both ECSP and a preceding BC did not have a higher EC‐specific risk of death (HR 0.80; 95% CI: 0.49–1.31; *p* = 0.373) compared to those chemotherapy recipients with EC as a first primary (Table 5). Differences in the proportion of ECSP histological subtypes and stage at diagnosis by most common first primary cancer site are noted in Table S1.

**TABLE 4:** Determinants of EC cause‐specific survival for ECSPs with first primary BC and CRC, Florida 2005–2016

Prognostic factors	HR (95% CI)	*p‐value*
Age category		
15–44 (*n* = 23)	REF	
45–54 (*n* = 168)	1.15 (0.14–9.40)	0.894
55–64 (*n* = 413)	1.29 (0.17–9.54)	0.806
65–74 (*n* = 557)	1.51 (0.21–11.08)	0.686
75+ (*n* = 620)	2.50 (0.34–18.18)	0.365
Race/ethnicity		
White (*n* = 1355)	REF	
Black (*n* = 178)	0.89 (0.56–1.42)	0.629
Hispanic (*n* = 218)	1.23 (0.80–1.90)	0.339
API (*n* = 21)	0.81 (0.10–6.49)	0.842
Other (*n* = 9)	2.01 (0.62–6.48)	0.245
Histology		
Type I		
Low‐grade endometrioid (*n* = 829)	REF	
Type II		
All histologies combined (*n* = 730)	5.95 (3.70–9.56)	<0.0001
High‐grade endometrioid (*n* = 219)	4.12 (2.31–7.34)	<0.0001
Carcinosarcoma (*n* = 173)	11.39 (6.68–19.41)	<0.0001
Clear cell (*n* = 39)	5.47 (2.44–12.25)	<0.0001
Mixed (*n* = 91)	5.71 (2.89–11.25)	<0.0001
Serous (*n* = 208)	4.93 (2.80–8.68)	<0.0001
Other (*n* = 222)	2.86 (1.54–5.31)	0.0008
ECSP stage		
Localized (*n* = 1072)	REF	
Regional (*n* = 415)	2.60 (1.77–3.83)	<0.0001
Distant (*n* = 129)	6.47 (4.11–10.19)	<0.0001
Unknown (*n* = 165)	6.22 (3.65–10.61)	<0.0001
First primary cancer site		
Breast (*n* = 1422)	REF	
Colorectal (*n* = 359)	1.47 (1.04–2.06)	0.028
First primary cancer site stage		
Localized (*n* = 775)	REF	
Regional (*n* = 368)	0.76 (0.54–1.09)	0.133
Distant (*n* = 50)	0.74 (0.30–1.88)	0.531
Unknown (*n* = 588)	1.27 (0.76–2.12)	0.369

Abbreviations: BC, breast cancer; EC, endometrial cancer; ECSP, endometrial cancer as a second primary; HR, hazard ratio.

**TABLE 5:** Determinants of EC cause‐specific survival for women with first primary EC and women with ECSP and first primary BC only, Florida 2005–2016

Prognostic factors	HR (95% CI)	*p‐value*
Age category		
15–44 (*n* = 2467)	REF	
45–54 (*n* = 6041)	0.95 (0.52–1.72)	0.864
55–64 (*n* = 13,315)	0.86 (0.49–1.50)	0.600
65–74 (*n* = 13,004)	1.17 (0.67–2.02)	0.587
75+ (*n* = 9308)	1.16 (0.66–2.05)	0.606
Race/ethnicity		
White (*n* = 31,999)	REF	
Black (*n* = 5031)	1.52 (1.23–1.89)	0.0001
Hispanic (*n* = 6088)	0.94 (0.71–1.24)	0.659
API (*n* = 523)	1.08 (0.47–2.44)	0.863
Other (*n* = 495)	2.73 (0.67–11.07)	0.161
Histology		
Type I		
Low‐Grade Endometrioid (*n* = 25,339)	REF	
Type II		
All histologies combined (*n* = 13,049)	1.87 (1.31–2.67)	0.0006
High‐grade endometrioid (*n* = 5560)	1.70 (1.13–2.58)	0.012
Carcinosarcoma (*n* = 2553)	2.52 (1.71–3.70)	<0.0001
Clear cell (*n* = 699)	2.53 (1.51–4.22)	0.0004
Mixed (*n* = 1541)	1.40 (0.90–2.17)	0.137
Serous (*n* = 2696)	1.64 (1.11–2.43)	0.014
Other (*n* = 5748)	2.02 (1.33–3.07)	0.001
Endometrial cancer stage		
Localized (*n* = 26,357)	REF	
Regional (*n* = 9360)	2.18 (1.38–3.44)	0.0008
Distant (*n* = 3243)	5.84 (3.56–9.58)	<0.0001
Unknown (*n* = 5176)	4.33 (2.62–7.16)	<0.0001
Breast cancer site stage		
Localized (*n* = 640)	REF	
Regional (*n* = 250)	1.22 (0.81–1.83)	0.348
Distant (*n* = 26)	1.28 (0.80–2.05)	0.309
Unknown (*n* = 506)	1.22 (0.79–1.90)	0.375
Chemotherapy treatment		
For first primary EC only (*n* = 6728)	REF	
For ECSP and first primary BC (*n* = 89)	0.80 (0.49–1.31)	0.373

Abbreviations: BC, breast cancer; EC, endometrial cancer; ECSP, endometrial cancer as a second primary; HR, hazard ratio.

## 4 DISCUSSION

This study brings to light new knowledge on ECSPs. These account for approximately 6% of all EC cases, are of worse histological type, and present at a more advanced stage in comparison to ECs as a first primary. Despite this, EC‐specific survival is higher for ECSPs than first primary ECs after taking into account these tumor‐related characteristics. Survival outcomes vary according to primary cancer site. Subsequent to BC, ECSP survival is higher than EC as a first primary. For ECSPs following CRC, survival was equal to that of ECs as a first primary, but worse than for those ECSPs following BC. Finally, chemotherapy for previous BC, which is often similar to that given for EC (e.g., anthracyclines), does not seem to affect the overall effectiveness of chemotherapy for ECSPs.

In absolute terms (i.e., 5‐year survival), ECSP survival is worse than that of ECs as a first primary. This is primarily explained by older age, more advanced stage of disease, and a higher proportion of Type II ECs (i.e., serous, carcinosarcoma, clear cell, mixed‐cell, and high‐grade endometrioid) which are associated with worse survival^26^, ^27^ among ECSPs. However, after taking into account these clinical characteristics, ECSPs have improved survival relative to ECs as a first primary. The overall evidence in this study points toward a beneficial role of being under healthcare surveillance/follow‐up for a previous primary which may enable better and more timely management of the disease. Moreover, being under healthcare surveillance diminishes survival disparities. For instance, the impact of age and race/ethnicity (for Blacks and Hispanics) on survival is marked among EC as a first primary^27^, ^36^ but disappears when the analysis is restricted to ECSPs. From a health disparities standpoint, these results are indicative of the importance of regular ongoing gynecological surveillance in the population at large which could potentially mitigate the currently established racial/ethnic disparities present for EC as a first primary.

There are some notable differences according to first primary cancer site. Women with a BC‐ECSP combination have improved survival in comparison to those with a first primary EC, after adjusting for all relevant sociodemographic and tumor‐related characteristics. This finding is similar to Matsuo et al. 2019 which found that women with uterine cancer who had antecedent BC were 30% less likely to die from uterine cancer.^37^ Given the common hormonal mechanism with BC and EC, this may also be indicative of differences in hormone therapy responsiveness. Prior research has suggested that tamoxifen‐related EC constitutes a subset of more favorable molecular and clinical profiles^38^, ^39^ which may partly account for the observed survival advantage also observed in our study. However, controversy remains on this topic as other studies have demonstrated a higher risk of mortality^40^ and more aggressive histological subtypes^41^ for tamoxifen‐treated women with EC.

ECSPs with antecedent CRC have worse survival than those with BC in multivariable analysis. Although ECSPs with a first primary BC have a higher proportion of Type II histological subtypes, those with first primary CRC have a larger distribution of other miscellaneous histology types (e.g., undifferentiated) (Table S1). CRC‐ECSPs in this subset have an older age at diagnosis and a more advanced stage at diagnosis for both the ECSP and first primary CRC while BC‐ECSPs are more often diagnosed at localized stage (Table S1). Previous studies have shown that patients with ECSPs after radiotherapy for CRCs have worse survival compared to those with a first primary EC only.^13^ However, only 12% of patients (*n* = 42) with first primary CRC received radiotherapy in our sample, so we could not assess the impact of this previous treatment on survival of ECSP. Additionally, Lynch Syndrome patients only account for a small proportion of CRC and EC cases, so it is unlikely that their tendency to have tumors with a more aggressive clinical course^42^ would impact our results. Given our findings, CRC female survivors may constitute an at‐risk population for EC for whom long‐term gynecological follow‐up is warranted and emphasizes the importance of delivering risk‐based healthcare for CRC survivors as outlined by the American Cancer Society's Colorectal Cancer Survivorship Care Guidelines.^43^

Lastly, radiation, chemotherapy, and/or targeted therapy agents may alter future disease biology. This in effect impacts subsequent treatment options and outcomes. In this context, there was an interest in assessing how repeated chemotherapy treatment for a first primary BC and subsequent ECSP could impact the survival of the ECSP. Our study findings suggest that prior chemotherapy treatment for BC does not seem to affect the overall therapeutic effect of chemotherapy treatment for ECSPs.

There are several strengths to be noted in our study. To our knowledge, this is the first study to examine ECSP survival for first primary cancers of all sites. It is a population‐based study and by including all cases of EC, we avoid selection bias related to health care access and referral that are commonly present in hospital data series. This study uses the experience of women from Florida, a very diverse population, that may be considered representative of the larger US racial/ethnic groups: Whites, Blacks, and Hispanics. However, some limitations cannot be overlooked. FCDS, as is the case with other registries, is limited in terms of clinical and risk factor data (e.g., obesity, diet, smoking) which can influence the development and survival for second cancers. We had no specific information on treatment or genetic‐related factors such as receipt of multi‐year therapies (i.e., Tamoxifen) or data on Lynch Syndrome diagnosis and *BRCA* gene mutations that may constitute specific ECSP subgroups with unique survival patterns. Moreover, data on more novel therapies such as tyrosine kinase inhibitors of which the long‐term impact and relationship with second primaries are unknown, was unavailable. Lastly, we did not have access to comorbidity data. However, the effect of this specific limitation is greatly diminished by our choice of cause‐specific survival (EC‐specific) rather than all‐cause survival as our outcome of interest. We utilized a cause‐specific approach that uses time‐to‐event data and treats competing events (including deaths by other causes) as censored observations rather than a competing risk approach which accounts for the chance of dying from the EC among those who are event free and among those who experienced a competing event. In the former, the risk set includes only those who are free of the event‐of‐interest (i.e., death due to EC) while for the latter, the risk set includes those who are event free as well as those who experienced a competing risk (death due to cause other than EC). To rule out a potential impact on survival by deaths from other causes, we conducted a competing risk analysis using the Fine and Gray sub‐distribution hazard regression modeling approach to estimate cumulative incidence rates of death from EC over time, with death from other cause as the competing risk.^44^ Similar results (data not shown) were obtained; thus, ruling out the possibility that our findings were due to not accounting for competing risks of death in the standard Cox‐proportional hazard statistical analyses.

As the cancer survivor population increases and the US population ages, it is imperative to study second primary malignancies in order to better understand their causes, develop prevention strategies, and ensure effective treatments. Currently, patients with active secondary malignancies and prior cancer history are commonly excluded from clinical trials,^5^ and therefore population‐based studies, such as this one, are an important source of data. Examining ECSPs provides insight into the importance of primary prevention of subsequent malignancies among cancer survivors.^45^, ^46^ ECSPs have improved survival in relation to EC as a first primary which can be seen as a success of current healthcare practices including follow‐up care. Nevertheless, there is heterogeneity in ECSP survival according to the primary site, notably rendering those with first primary CRC as a population of interest. Additionally, there is no evidence of diminished survival after use of repeated chemotherapy for successive cancers. Finally, our study suggests that improvements in aligning registry definitions of second primaries with clinical practice (clinically independent second primaries) could be beneficial in order to provide further insight into the etiology and clinical management of subsequent malignancies and public health efforts needed among cancer survivors. More research elucidating the complex profiles of second primary cancers is warranted.

## CONFLICT OF INTEREST

The authors declare no potential conflicts of interest.

## AUTHOR CONTRIBUTIONS

Heidy N. Medina: Conceptualization, Formal analysis, Visualization, Writing ‐ Original Draft, Writing ‐ Review and Editing. **Matthew P. Schlumbrecht**: Formal analysis, Writing‐ Review and Editing. **Frank J. Penedo**: Formal analysis, Writing‐ Review and Editing. **Paulo S. Pinheiro**: Conceptualization, Methodology, Formal analysis, Writing ‐ Original Draft, Writing‐ Review and Editing, Supervision.

## Supporting information


Table S1

## Data Availability

The authors confirm that, for approved reasons, some access restrictions apply to the data underlying the findings. These data are confidential public health records with personal identifiers that can only be released for specific use upon approvals from the Florida Department of Health Cancer Registry Program, Florida Department of Health Bureau of Vital Statistics, and the Florida Department of Health Institutional Review Board. These data are never available for public repository given the confidential information they contain. The datasets are available by request with required approvals from the Florida Department of Health Cancer Registry Program and Florida Department of Health Institutional Review Board. Applications for data request are available from the FCDS Webpage (http://fcds.med.miami.edu/inc/datarequest.shtml). The authors confirm that they did not have any special access privileges that others would not have.
