# Supplementary material for: Survival for endometrial cancer as a second primary malignancy
Source: Cancer Med. 2022 Jan 30;11(6):1490–501. doi: 10.1002/cam4.4554 (PMC8921898; doi:10.1002/cam4.4554)
Supplement: Supplementary file 1 — Table S1 [file CAM4-11-1490-s001.docx]

| **Supplementary Table 1. Clinical and Demographic Characteristics of ECSPs following First Primary BC and CRC. Florida 2005-2016.** | | | | | |
| --- | --- | --- | --- | --- | --- |
|  | | **Total**^†^  **n (%)** | **ECSP-First Primary BC**  **n (%)** | **ECSP-First Primary CRC**  **n (%)** | ***P-value^§^*** |
| Total | | 2,879 (100.0%) | 1422 (49.4%) | 359 (12.5%) |  |
| Median Age at Diagnosis of ECSP (years) | | 69.0 | 69.0 | 72.0 |  |
| Median Time of Diagnosis of ECSP following First Primary (months) | | 40.8 | 49.2 | 43.9 |  |
| ECSP Histology | |  |  |  | 0.041 |
| Type I | Low-Grade Endometrioid | 1334 (46.3%) | 663 (46.6%) | 166 (46.2%) |  |
| Type II | All Histologies Combined | 1101 (38.2%) | 597 (42.0%) | 133 (37.1%) |  |
|  | High-Grade Endometrioid | 342 (11.9%) | 178 (12.5%) | 41 (11.4%) |  |
|  | Carcinosarcoma | 253 (8.8%) | 144 (10.1%) | 29 (8.1%) |  |
|  | Clear Cell | 66 (2.3%) | 30 (2.1%) | ‡ |  |
|  | Mixed | 134 (4.7%) | 81 (5.7%) | ‡ |  |
|  | Serous | 306 (10.6%) | 164 (11.5%) | 44 (12.3%) |  |
| Other | | 444 (15.4%) | 162 (11.4%) | 60 (16.7%) |  |
| ECSP Stage | |  |  |  | 0.029 |
| Localized | | 1667 (57.9%) | 878 (61.7%) | 194 (54.0%) |  |
| Regional | | 644 (22.4%) | 319 (22.4%) | 96 (26.7%) |  |
| Distant | | 259 (9.0%) | 99 (7.0%) | 30 (8.4%) |  |
| Unknown | | 309 (10.7%) | 126 (8.9%) | 39 (10.9%) |  |
| First Primary Cancer Site Stage | |  |  |  | <.0001 |
| Localized | | 1119 (38.9%) | 640 (45.0%) | 135 (37.6%) |  |
| Regional | | 525 (18.2%) | 250 (17.6%) | 118 (32.9%) |  |
| Distant | | 242 (8.4%) | 26 (1.8%) | 24 (6.7%) |  |
| Unknown | | 993 (34.5%) | 506 (35.6%) | 82 (22.8%) |  |
| †. Total includes other first primary sites not shown here; ‡ Not reported; observations fewer than 10; §. *P-value* from chi-square test  *Abbreviations: ECSP, Endometrial Cancer as a Second Primary; BC, Breast Cancer; CRC, Colorectal Cancer* | | | | | |
